# Prolonged exposure to simulated microgravity diminishes dendritic cell immunogenicity

**DOI:** 10.1038/s41598-019-50311-z

**Published:** 2019-09-25

**Authors:** Nichole Tackett, Jillian H. Bradley, Emily K. Moore, Stefanie H. Baker, Stephanie L. Minter, Brian DiGiacinto, Jennifer P. Arnold, Randal K. Gregg

**Affiliations:** 1 Department of Biology, Chemistry and Physics, Converse College, Spartanburg, SC 29302 USA; 2 Magnolia Research Center, Division of Biomedical Sciences, Department of Microbiology and Immunology, Edward Via College of Osteopathic Medicine Carolinas Campus, Spartanburg, SC 29303 USA; 3 Department of Biology, Wofford College, Spartanburg, SC 29303 USA; 4 Department of Basic Medical Sciences, DeBusk College of Osteopathic Medicine at Lincoln Memorial University-Knoxville, Knoxville, TN 37932 USA

**Keywords:** Antigen-presenting cells, T-helper 1 cells, Cytotoxic T cells, Immunosuppression, Cell death and immune response

## Abstract

Immune dysfunction due to microgravity remains a hurdle in the next step of human space exploration. Dendritic cells (DC) represent a critical component of immunity, given their role in the detection of invaders and the subsequent task of activating T cells to respond and eliminate the threat. Upon encounter with microbes, DC undergo a process of maturation, whereby the cells upregulate the expression of surface proteins and secrete cytokines, both required for the optimal activation of CD4^+^ and CD8^+^ T cells. In this study, DC were cultured from 2–14 days in a rotary cell culture system, which generates a simulated microgravity (SMG) environment, and then the cells were assessed for maturation status and the capacity to activate T cells. Short-term culture (<72 h) of DC in SMG resulted in an increased expression of surface proteins associated with maturation and interleukin-6 production. Subsequently, the SMG exposed DC were superior to Static control DC at activating both CD4^+^ and CD8^+^ T cells as measured by interleukin-2 and interferon-γ production, respectively. However, long-term culture (4–14 d) of DC in SMG reduced the expression of maturation markers and the capacity to activate T cells as compared to Static DC controls.

## Introduction

Since the beginning of space exploration, it has been clear that spaceflight promotes changes in the balance between the immune system and the resident microbial population of the human body. Indeed, a number of opportunistic infections have been reported in otherwise healthy astronauts either during or following spaceflight^1–5^. Development of opportunistic infections has long since been related to a weakened immune system. Accordingly, aspects of spaceflight, in particular, weightlessness or microgravity, negatively impacts the function of immune cells^6^. The host response depends upon specialized cells, called T cells, to control and eliminate disease-causing microbes as well as cancer cells. Upon recognition of specific microbial or tumor peptides, the T cells are activated to expand and differentiate into a population of effector T lymphocytes. A study, conducted during a Space Shuttle mission, demonstrated that while viral-specific T cell numbers were not significantly altered during spaceflight, T cell functions were decreased and the deficit persisted up to 14 days following the return of the astronauts to Earth^6^.

To date, few studies have examined the effect of microgravity upon dendritic cells (DC), the cells tasked with the function of displaying the required peptide for stimulation of the T cells. DC normally reside in tissues and lymphoid organs and continuously collect and process proteins (or antigens) from microbes and dying host cells (either infected, transformed or aged). DC cleave the proteins into peptides and attach them to molecules called major histocompatibility complexes (MHC). Subsequently, the MHC-peptide structures are transported to the surface of DC so that the peptide can be displayed for interaction with peptide-specific T cells^7^. In order for DC to optimally activate the T cells, DC must undergo a process of maturation. Conserved structures of microbes, such as cell wall lipopolysaccharide (LPS), can trigger dendritic cell maturation through the direct ligation of constitutively expressed Toll-like receptors (TLR). Subsequent signaling results in the surface expression of proteins such as MHC class I and II molecules, CD80, CD86, CD40, and the secretion of immune-modulating proteins called cytokines (*i*.*e*. interleukin (IL)-6, IL-12)^7^. MHC I- and MHC II-peptide complexes engage peptide-specific T cell receptors (TCR), expressed by CD8^+^ and CD4^+^ T cells, respectively. However, engagement of the TCR by peptide alone is insufficient to optimally activate the T cells. Mature DC express CD80 and CD86, which can bind to CD28, a constitutively expressed protein on the surface of T cells. Together with TCR signaling, the single activated T cell can now clonally expand into a large population of peptide-specific T cells. Yet, T cell acquisition of effector function can only be achieved by a third signaling event provided by the dendritic cell-derived cytokine (*i*.*e*. IL-4, IL-12 and others). Once the cytokine binds the cytokine receptor on the T cell, the activated T cell expresses genes associated with effector functions. Effector CD4^+^ T cells produce cytokines that direct the responses of other cells in the body, including CD8^+^ T cells or cytotoxic T lymphocytes. Effector CD8^+^ T cells can produce cytokines and cytotoxins that act directly to destroy pathogenic microorganisms and cancer cells. Aberrations of the T cell activation process can lead to successful colonization of the infectious agent or cancer cells, dissemination to other sites within the body and disease^8,9^. Importantly, space radiation and cancer serve as one of the largest hurdles for long-term human residence and exploration beyond the protective magnetic field of the Earth. Despite the use of current radiation shielding technologies, excessive exposure of astronauts to radiation is inevitable, and a functioning dendritic cell-T cell network may be critical for cost-effective research in space, preservation of astronaut health and overall success of each mission. Therefore, studies of dendritic cell viability and function in microgravity are imperative for all human spaceflight outside of Earth orbit.

Given the reports of T cell diminished responses, when exposed to microgravity environments^10,11^, we suspected that an underlying mechanism for the T cell impairment resided with the nature of dendritic cell-T cell interactions. Because of the large expense of spaceflight and limited opportunities for research in space, the National Aeronautics and Space Agency (NASA) developed and validated an Earth-bound system, rotary cell culture system (RCCS), that has facilitated studies pertaining to the effect of simulated microgravity (SMG) upon cell structure and function^12,13^. The RCCS is a type of clinostat, which rotates the cells in a manner that counters the gravitational force through rotation in one or two axes (*i*.*e*. clinorotation). The RCCS is used for culturing both anchorage dependent and suspension cells. Two culture approaches were developed for clinostats, the slow turning lateral vessel (STLV) and the high aspect ratio vessel (HARV)^13^. The STLV was designed, more specifically, to culture anchorage dependent cells in 3D clusters given its cylindrical shape. The HARV, used in this study, was created to culture suspension cells as well as 3D aggregates of anchorage dependent cells, given its disk shape. Cells are loaded into an HARV or rotation vessel (RV) in 10 ml of culture media and rotated at speeds that suspend the cells in a continuous state of free fall with the absence of large shear forces. The cells in the RCCS aggregate in 3D with high nutrient and oxygen transport, but unlike spinner flasks, mechanical damage is avoided. Additional clinostat devices are used for SMG studies involving organisms as well as suspension cells. For instance, a 2D clinostat has been used to grow plants at low speeds. A 3D clinostat can rotate organisms about two axes, perpendicular to the gravity vector. The 3D clinostat possesses two independently rotating frames that are mounted perpendicular to one another and operates with the frames running in the same direction at the same speed. However, when the two frames are operating with different speeds and direction, the system is termed a random positioning machine (RPM). Recently, surface proteins and signaling molecules, associated with T cell activation, were shown to be decreased within 60 min after culture in a 2D clinostat^14^. A decreased proliferation of T cells was noted in a separate study employing a 48 h culture in a 3D clinostat^15^. SMG can also be generated by magnetic levitation, which occurs when diamagnetic substances (water, organic solvents, etc.), cells and small organisms (frogs and grasshoppers) are placed in a strong magnetic field^16^. Given that SMG can occur instantaneous to placement within the field, this process allows for the assessment of rapid cellular processes. However, exposure of cells to high magnetic fields remains a concern with regard to controls for the use of magnetic levitation. RPM and magnetic levitation culture of A431 human epidermal cancer cells resulted in a morphology indistinguishable to that in real microgravity^16^. We chose to employ the RCCS, given that culture of cells in this system has demonstrated morphological, structural and functional changes similar to those observed after exposure to real microgravity^17^.

Using a murine dendritic cell line, JAWS II^18^, and bone marrow-derived DC (BMDC), we tested the effects of short-term (72 h) and long-term (up to 14 d) exposure to SMG upon dendritic cell maturation and capacity to activate peptide-specific CD4^+^ OT-II T cell hybridomas (TCH)^19^ and CD8^+^ OT-I T cells. Cell surface proteins, such as MHC II, CD80 and CD86, served as markers of maturation. Activation of T cells was determined by production of the cytokines, IL-2 and interferon-γ (IFN-γ). Our data indicated that short-term SMG culture of DC resulted in an increase in maturation status and T cell reactivity, however, long-term SMG culture diminished dendritic cell functions.

## Methods

### Cells

The murine JAWS II DC line was obtained from the American Type Culture Collection (ATCC, Manassas, VA, USA). JAWS II DC were sustained in alpha minimum essential medium (+ribonucleosides and deoxyribonucleosides) supplemented with 20% fetal bovine serum (FBS), 5 ng/ml recombinant murine GM-CSF (BD Biosciences, San Jose, CA, USA) and gentamicin (Sigma-Aldrich, St. Louis, MO, USA). The CD4^+^ TCH line, BO97.10.5, termed OT-II TCH, was a kind gift from Philippa Marrack, National Jewish Health, Denver, CO, USA. The OT-II TCH have a specific TCR (I-A^b^-restricted) that recognizes chicken ovalbumin (OVA) peptide, amino acids 323–339 or OVA323-339 (InvivoGen, San Diego, CA, USA). OT-I CD8^+^ T cells (H2-K^b^-restricted) were generated from spleens and lymph nodes harvested from 8- to 12-week-old OT-I TCR transgenic C57Bl/6 mice (Charles River Laboratories, Wilmington, MA, USA). Briefly, upon delivery, the cells were harvested from the animals and then stimulated with IL-2 (50 ng/ml) (R&D Systems, Minneapolis, MN, USA) and 10 μg/ml of OVA peptide, amino acids 257–264 or OVA257-264 (InvivoGen), in complete medium containing RPMI-1640 (ATCC) with 10% FBS, 50 μM β2-mercaptoethanol (Sigma Aldrich) and gentamicin for 3 days and subsequently cryopreserved in liquid nitrogen^20^. When used in experimentation, the OT-I CD8^+^ T cells were thawed in complete medium + IL-2 (25 ng/ml), cultured for two days and then replenished with media and IL-2 for two further days prior to testing. At day 7, purity of the CD8^+^ T cell population was consistently 95% as measured by flow cytometry (data not shown). BMDC were generated from the marrow of 8- to 12-week-old C57Bl/6 mice (Charles River)^21^. Briefly, femurs were isolated from the mice and marrow expelled using a syringe and the harvested cells were counted and cryopreserved in liquid nitrogen^22^. To generate BMDC, bone marrow was thawed in 10 ml of complete medium composed of RPMI-1640 with 10% FBS, 20 ng/ml recombinant murine GM-CSF and gentamicin for 3 days in a 90 mm Petri dish. On day 3, an additional 10 ml of fresh complete medium was added to the Petri dish with 20 ng/ml of GM-CSF. On day 6, the cells in suspension and loosely adherent cells represent the differentiated BMDC. Purity of the immature BMDC at day 6 was 85% by flow cytometry (data not shown). All cells were maintained at 37 °C in a humidified atmosphere of 5% CO_2_ and 95% air. C57Bl/6 and OT-I TCR transgenic mice were processed the day of arrival to the housing facility at Converse College and all procedures were approved and performed in accordance with the guidelines and regulations established by the Institutional Animal Care and Use Committee.

### Culture in the rotary cell culture system (RCCS)

The RCCS (Synthecon Inc., Houston, TX, USA) generates an SMG environment for cell culture and is composed of the clinorotation device and speed control unit^13^. First, cells (2 × 10^5^/ml) were placed in a 10 ml rotation vessel (RV) containing the appropriate complete medium. Following air removal, the RV was attached vertically to the clinorotation device, which was placed within the CO_2_ incubator. The speed control unit was adjusted to rotate the cells at 16 rpm. During rotation, cell suspension was uniform with the cells remaining in the center of the RV where shear forces are at a minimum. JAWS II DC or BMDC were added to an RV and rotated between 2–14 days with media exchange every two days. Given that the DC are anchorage dependent, 5 mg/ml of Cytodex-3 microcarrier beads (Sigma Aldrich) can be added to the cultures prior to rotation in order to maintain viability. To maintain healthy growth of the cells, a small sample was examined by microscopy to determine if an adjustment to cell concentration was necessary during media replenishment. Cells cultured in the RCCS were considered growing in simulated microgravity (SMG) conditions. Controls for the experiments involve stationary culture of JAWS II DC or BMDC in 25 cm^2^ flasks (10 ml total volume including beads) under normal conditions within the incubator. Flasks were chosen for control growth as preliminary experiments indicated that the use of a 25 cm^2^ flask yielded no significant difference in assay outcomes when compared to stationary culture of cells in an RV. Cells cultured in the flasks were considered growing in Static conditions.

JAWS II DC (or BMDC) cultured in Static or SMG conditions were designated in all experimentation as Static JAWS II DC (or BMDC) and SMG JAWS II DC (or BMDC), respectively.

### DC signaling and surface activation markers

For assessment of cell signaling events and expression of surface protein markers of maturation, Static and SMG JAWS II DC and BMDC were incubated with manufacturer recommended concentrations of corresponding fluorochrome-conjugated antibodies. Subsequently, 50 × 10^3^ events were collected on a Millipore Guava Cytometer (EMD Millipore, Billerica, MA, USA) and analyzed using GuavaSoft 2.7 software. At the indicated time point, DC, both non-adherent and adherent, were harvested from the RVs (SMG) and culture flasks (Static) and separately incubated with 0.25% Trypsin-EDTA (ATCC) to detach cells from the beads. Then the cells were counted and added to wells of a round-bottom 96 well assay plate at 100 × 10^3^ cells/well. Following a 20 min incubation with FcBlock (BD Biosciences), fluorochrome-conjugated antibodies against the following murine protein targets were added to the Static and SMG DC as described in the figures: pSTAT-5-FITC (SRBCZX, eBiosciences, San Diego, CA, USA), pERK1/2-APC (MILAN8R, eBiosciences), p-mTOR-PE (MRRBY, eBiosciences), activated caspase-3-PE (C92-605, BD Biosciences), GM-CSFR (MP1-22E9, BioLegend, San Diego, CA, USA), MHC I-APC (AF6-88.5.5.3, eBiosciences), MHC II-PECy5 (M5/114.15.2, eBiosciences), CD40-PE (1C10, eBiosciences), CD80-PE (16-10A1, BD Biosciences), CD86-APC (GL1, eBiosciences), 4-1BBL (or CD137L)-PE (TKS-1, eBiosciences) and DC-SIGN (or CD209)-PE (SH10, eBiosciences). Prior to the addition of fluorochrome-conjugated antibodies against intracellular signaling molecules, dendritic cell membranes were permeabilized with Cytofix/Cytoperm solution (BD Biosciences). In addition, some cells were labeled with the following isotype control antibodies: hamster IgG-PE (eBio299Arm, eBiosciences), rat IgG2A-PE (eBR2a, eBiosciences), rat IgG2A-APC (eBR2a, eBiosciences) and rat IgG2B-PECy5 (eB149/10H5, eBiosciences). Following a 30 min incubation of the cells at 4 °C, the cells were washed, fixed with 2% formaldehyde (20 min at room temperature), and then analyzed by flow cytometry.

For all of the flow cytometric measurements above, the mean fluorescence intensity (MFI) was obtained during data analysis and plotted as bar graphs. For each protein assessed, MFI for the Static and SMG DC were plotted and the isotype value omitted. Of note, when assessing maturation status of JAWS II DC, cultured in SMG or Static conditions for 72 h, MFI was plotted for the unstimulated DC only, given stimulation by cytokines just enhanced an existing significant difference.

### T cell activation assays

Following incubation of JAWS II DC (3, 7, 12 or 14 d) or BMDC (2 d) in Static or SMG conditions, the cells were harvested using Trypsin as given above and plated at 10 × 10^3^ DC/well in a round bottom 96 well assay plate. OT-II TCH (50 × 10^3^ cells/well) were added to the wells containing the Static or SMG JAWS II DC with or without either 1 mg/ml of full length ovalbumin (OVA protein, Sigma-Aldrich) or 0.1 mg/ml of OVA323-339. Cytokine production of OT-II TCH, in the presence of JAWS II DC alone, was omitted given these co-cultures yielded no background cytokine production^11^. Similarly, OT-I CD8^+^ T cells (50 × 10^3^ cells/well) were added to wells containing either Static or SMG BMDC with or without 10 μg/ml of OVA257-264. The above co-culture assays were incubated for 24 h in Static conditions and then supernatant collected for cytokine measurement.

For assessment of the T cell response to OVA323-339, as presented by DC within SMG, both T cells and DC were co-cultured in the RV and control flasks (10 ml total volume). First, the DC were harvested following 3 d culture in Static and SMG conditions, then washed, counted and placed into separate RV and 25 cm^2^ flasks at a concentration of 5 × 10^5^ JAWS II DC. Next, 2.5 × 10^6^ OT-II TCH and 0.1 mg/ml of OVA323-339 were added to the DC in the RV and control flasks and incubated in the indicated condition for 24 h. For the T cell responses to OVA protein, the Static and SMG JAWS II DC were harvested on day 3, washed, and refreshed with media containing 2 mg/ml of OVA protein and placed into the RV and 25 cm^2^ flasks with the T cells as discussed above for 24 h. During this time, OVA protein would undergo proteolysis, OVA323-339 liberated and displayed on MHC class II molecules for OT-II TCH recognition.

### Detection of IL-2 and IFN-γ in cell cultures

IL-2 and IFN-γ production by T cells were detected using BD Biosciences recommended ELISA protocols. IL-2 and IFN-γ from cell supernatants were captured by rat anti-mouse IL-2 (JES6-1A12, BD Biosciences) and rat anti-mouse IFN-γ (R4-6A2, BD Biosciences), respectively. Detection of the captured cytokines was accomplished through the use of biotinylated rat anti-mouse IL-2 (JES6-5H4, BD Biosciences) and biotinylated rat anti-mouse IFN-γ (XMG1.2, BD Biosciences) antibodies. Graded amounts of recombinant murine IL-2 and IFN-γ were included for generation of standard curves from which concentrations were extrapolated using the linear portion of each curve. Reported IL-2 and IFN-γ values were acquired through the use of a BioTek Eon microplate spectrophotometer (BioTek Instruments Inc., Winooski, VT, USA) and analyzed using Gen 5 software version 2.01.14.

### Statistical analyses

Results were expressed, as noted in each figure caption, with bar graphs generally represented as means of duplicate to triplicate experiments and each test sample performed in a range of n = 3-10. Differences between group means were calculated for statistical significance using the unpaired *t* test with p < 0.05 considered as a significant difference and denoted with a single asterisk (*). All *t* test calculations were performed using GraphPad software (GraphPad Software Inc., La Jolla, CA, USA).

## Results and Discussion

### Signal transduction and cell number of JAWS II DC is altered by SMG

**Figure 1:**
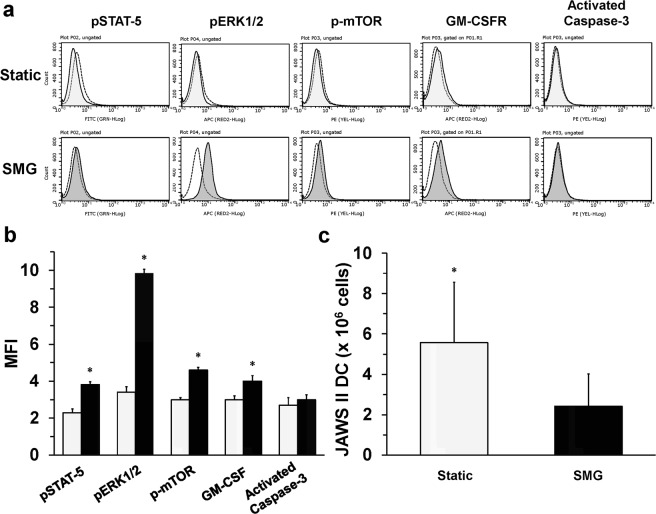
SMG promotes signaling in unstimulated JAWS II DC. Unstimulated JAWS II DC (2 × 10^5^/ml) were cultured in Static (light gray) or SMG (dark gray) conditions in media for 48 h or 72 h. (**a**) DC were collected after 48 h of culture, permeabilized and then stained with antibodies for pSTAT-5, pERK1/2, p-mTOR and activated caspase-3 and their corresponding isotype controls (open histogram/broken line). GM-CSFR detection was accomplished with antibody in non-permeabilized cells. (**b**) Graph represents the mean fluorescence intensity (MFI) of each of the molecules examined in (a) for Static (light gray bars) and SMG DC (black bars). One representative experiment of two independent experiments with similar results is shown. (**c**) Following 72 h of culture in Static (light gray bar) or SMG (black bar) conditions, the cells were enumerated by trypan blue exclusion. Bar graph represents the means of independent cultures (n = 16) + SD of all samples. In (b), *p-value ≤ 0.05 comparing the intracellular and surface expression of molecules by SMG and Static DC. In (c), *p-value ≤ 0.05 comparing the counts of Static and SMG DC.

Previously, signaling pathways, such as NF-κB and MAPK, were reported to be modified in T cells exposed to SMG, generated by an RPM^23^. Aberrations of signaling resulted in altered gene expression, which impacted T cell activation. We sought to investigate whether SMG would have a similar effect upon relevant dendritic cell signaling molecules. Both JAWS II DC and BMDC require the addition of granulocyte-macrophage colony-stimulating factor (GM-CSF) for growth *in vitro*. In fact, GM-CSF serves as the primary growth and differentiation factor of DC. Ligation of the GM-CSF receptor (GM-CSFR) results in activation of the following pathways: JAK2/STAT-5, PI3K/PKB (mTOR), MEK/ERK (MAPK) and NF-κB^24^. Phosphorylation of STAT-5 (pSTAT-5) serves as the key differentiation factor in the generation of DC from monocytes^25,26^. Additionally, pSTAT-5 is critical for dendritic cell proliferation and the capacity to stimulate T cells (immunogenicity). The major role of PI3K/PKB signaling is in the expansion and survival of cells differentiating into DC, however, survival of terminally differentiated DC is independent of PI3K^27^. Signaling through the MAPK pathway appears to contribute to the survival and differentiation of DC^24^. GM-CSFR-induced canonical NF-κB activation promotes survival, differentiation and immunogenicity of DC. Since pSTAT-5 can promote noncanonical activation of NF-κB to promote the immunogenicity of DC, pSTAT-5 was assessed for differential expression between Static and SMG JAWS II DC. Following a 48 h culture, SMG JAWS II DC expressed an increase in the intracellular levels of pSTAT-5 as compared to Static DC (Fig. 1a,b). Likewise, mTOR (p-mTOR) was increased in JAWS II DC, cultured in SMG compared to Static DC. The dendritic cell signaling pathway most impacted by SMG was ERK1/2 (pERK1/2) with an MFI more than three times that of Static DC (Fig. 1a,b). Upon SMG culture, JAWS II DC demonstrated a higher surface expression of GM-CSFR, which may account for the increased activation of these relevant signaling pathways, especially in the presence of GM-CSF. From the findings, we hypothesized that SMG JAWS II DC would likely demonstrate an increase in proliferation as compared to Static JAWS II DC. To this end, JAWS II DC were enumerated following 72 h culture in SMG and Static conditions. From 16 independent cultures, a consistent 2:1 ratio of Static to SMG DC was observed (Fig. 1c). Thus, SMG does not promote proliferation of the JAWS II DC. Interestingly, certain populations of immune cells undergo apoptosis upon culture in SMG, according to prior studies^2,4^. However, SMG JAWS II DC did not demonstrate a relevant level of activated caspase-3 when compared to Static DC (Fig. 1a). Indeed, neither population of DC was undergoing apoptosis following the 48 h culture. Similar results were obtained with 72 h dendritic cell cultures as well (data not shown). Most likely, JAWS II DC undergo slowed or arrested cell cycling when exposed to SMG. Overall, SMG increased intracellular signaling in unstimulated JAWS II DC, possibly through an enhanced expression of cell surface GM-CSFR and subsequent response to the exogenously added cytokine. While these pathways are associated with cellular expansion, the number of viable SMG DC were half that of Static cultured DC.

### SMG promotes a maturation phenotype in DC

**Figure 2:**
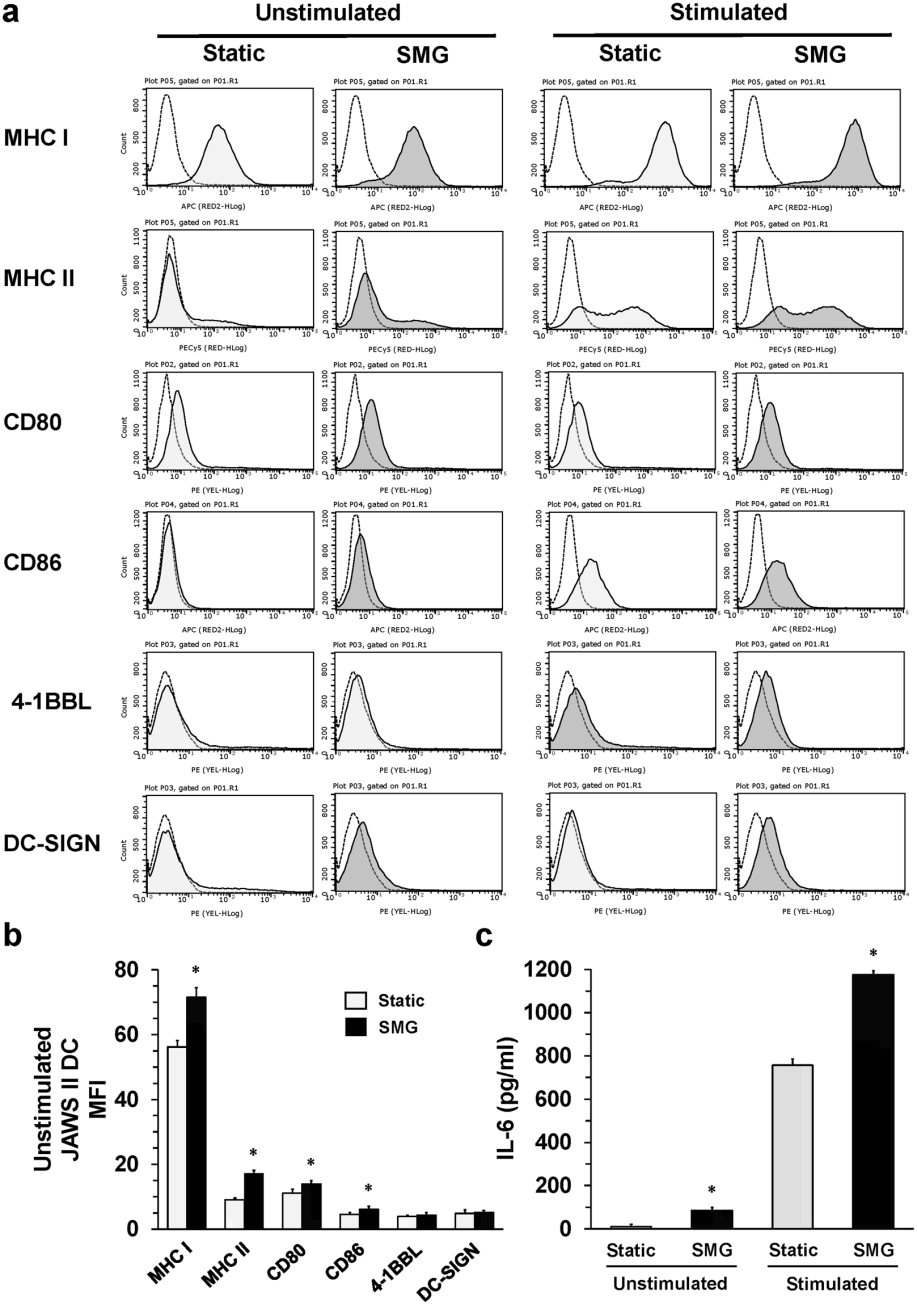
JAWS II DC undergo maturation when cultured in SMG. (**a**) Unstimulated and stimulated JAWS II DC (2 × 10^5^/ml) were cultured in Static (light gray) or SMG (dark gray) conditions for 72 h. JAWS II DC activated during Static or SMG culture were either incubated with (stimulated) or without (unstimulated) a cocktail of cytokines (IFN-γ, IL-4 and TNFα). Afterwards, the cells were collected and stained with antibodies for MHC class I and II, CD80, CD86, 4-1BBL and DC-SIGN and their corresponding isotype controls. Open histograms with broken lines represent isotype controls. (**b**) Graph represents the MFI of each of the surface molecules examined in (**a**) for unstimulated Static (light gray bars) and SMG JAWS II DC (black bars). (**c**) Culture supernatants, collected from unstimulated and stimulated JAWS II DC (2 × 10^5^/ml) cultured as in (**a**), were assessed for IL-6 production by ELISA. One representation of two independent experiments with similar results is shown for (**a**,**b**). For (**c**), the data represents the mean + SD of quadruplicates of two independent experiments. In (**b**), *p-value ≤ 0.05 comparing the expression of surface molecules by SMG and Static JAWS II DC. In (**c**), *p-value ≤ 0.05 comparing the production of IL-6 by unstimulated and stimulated SMG to Static JAWS II DC.

The primary function of DC is to capture and process proteins in order to present peptides directly to the T cells for activation of the adaptive immune response. In order to accomplish this task, DC must undergo a process of maturation to increase or induce expression of surface MHC class I and II molecules (signal 1), costimulatory ligands for T cells, CD80, CD86 and 4-1BBL (signal 2), and cytokine production (signal 3). All three signals are required for optimal stimulation of T cell proliferation and differentiation into effector T cells^4^. During infection, maturation stimuli for DC are provided either by ligation of Toll-like receptors, with conserved regions of microbial components, or signaling mediated through cytokine-cytokine receptor interactions. Unstimulated or immature DC employ macropinocytosis, phagocytosis and endocytosis as well as an array of C-type lectin receptors, such as DC-SIGN, for protein (antigen) capture^28,29^. Expression of DC-SIGN can increase upon dendritic cell maturation, thereby implicating its surface upregulation as a marker for immature to mature transition. Culture of unstimulated JAWS II DC in SMG resulted in an increase in DC-SIGN surface expression as compared to Static DC, however, the difference did not reach significance (Fig. 2a,b). Immature JAWS II DC can be induced to undergo maturation using a cocktail of stimulatory cytokines including IFN-γ, IL-4 and TNFα^18^. DC-SIGN expression increased in both cytokine-stimulated Static and SMG JAWS II DC, but significantly more DC-SIGN was detected on the cells cultured in SMG (data not shown, *p* < 0.05). Interestingly, DC-SIGN can also serve an important role in adhesion of DC to T cells for proper antigen display and recognition, and contribute to the costimulation (signal 2) of T cell activation. Thus, short-term culture of DC in SMG may enhance the capacity of the cells to capture antigen and activate T cells when compared to Static DC, especially when the DC are stimulated by maturation factors.

MHC class I molecules and CD80 are constitutively expressed on the cell surface of immature DC^30^. Indeed, unstimulated JAWS II DC were positive for both MHC class I and CD80 (Fig. 2a). However, unstimulated SMG JAWS II DC exhibited a significant increase in the expression of both of these proteins (Fig. 2a,b). In addition, SMG JAWS II DC expressed higher levels of MHC class II molecules and CD86 compared to Static JAWS II DC. Upon activation of the immature JAWS II DC with the cytokine cocktail, MHC class I and II molecules, CD80 and CD86 were all significantly increased (*p* < 0.05) on the surface of the SMG DC relative to the Static DC (Fig. 2a, stimulated). Therefore, SMG may serve as a type of stress that acts upon DC, similar to a microbial infection, to promote the maturation of DC. This outcome could be detrimental to human health over prolonged periods. That is, microgravity could induce maturation of DC without provocation. Given DC undergo apoptosis at some time point following maturation, loss of a large percentage of mature DC may put the astronaut at great risk for disease including opportunistic infections. To date, a number of cytokines and cellular interactions are known to trigger apoptosis in mature DC, however, the fate of these cells *in vivo* remains unclear^31^. Interestingly, all of the signaling molecules examined earlier (Fig. 1) can serve both as pro-apoptotic and anti-apoptotic factors. Since SMG enhanced their expression without resulting in apoptosis, likely SMG produced an overall anti-apoptotic signal, at least in the short-term. This provides a suggestion as to why fewer DC were recovered from SMG as compared to Static conditions (Fig. 1c). That is, cell cycle arrest can trigger apoptosis unless anti-apoptotic factors prevent pathway activation. A future study will examine the life span of SMG-activated DC.

**Figure 3:**
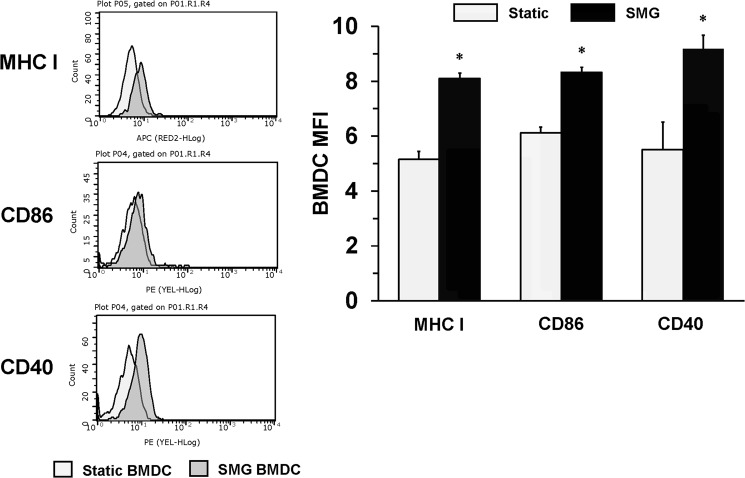
SMG upregulates maturation markers of BMDC. Freshly isolated BMDC were cultured in Static (light gray) or SMG (dark gray) conditions for 48 hours and assessed for the expression of the maturation markers, MHC class I, CD40 and CD86, by flow cytometry. The bar graph represents the MFI of each of the surface molecules examined for Static (light gray bars) and SMG (black bars) BMDC. The data represents the mean + SD of quadruplicates of two independent experiments. In the right panel, *p-value ≤ 0.05 comparing the expression of surface molecules by SMG and Static BMDC.

Since JAWS II DC are immortalized cells and subject to unchecked replication, we tested whether markers of maturation would be expressed in freshly differentiated murine bone marrow-derived DC (BMDC). To this end, immature BMDC were similarly cultured in Static and SMG conditions for 48 h and assessed for MHC class I, CD40 and CD86 surface expression. SMG BMDC also demonstrated a similar significant increase in expression of surface proteins related to a mature phenotype as compared to Static BMDC (Fig. 3). Therefore, SMG can promote the expression of proteins associated with signals 1 and 2, which operate to activate T cells.

In order for T cells to acquire appropriate effector functions, the DC must produce cytokines (signal 3). JAWS II DC have been shown to produce IL-6 upon stimulation with a cytokine cocktail^18^. To determine if SMG effects cytokine production by JAWS II DC, we measured the production of IL-6 by JAWS II DC when the cells were cultured in both SMG and Static conditions after 72 h. Surprisingly, SMG JAWS II DC secreted a significant amount of IL-6 in the absence of the cytokine stimulus (Fig. 2c). Activation with the cytokine cocktail enhanced production of IL-6 by SMG DC more than 10 times that of unstimulated SMG DC and nearly 2-fold compared to stimulated Static DC. Together, SMG exposure activates the maturation process of DC resulting in the expression of molecules responsible for the capture and presentation of antigens for T cell expansion and cytokines to facilitate acquisition of effector functions.

### SMG DC effectively activates antigen-specific T cells

**Figure 4:**
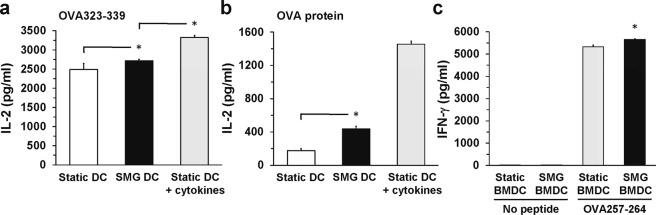
SMG DC are more effective in the activation of antigen-specific T cells than Static DC. JAWS II DC or BMDC (2 × 10^5^/ml) were cultured in Static (white and gray bars) or SMG (black bar) conditions for 72 and 48 hours, respectively. Some JAWS II DC (+cytokines) were incubated with a cocktail of cytokines (IFN-γ, IL-4 and TNFα) 6 hours prior to harvest. Subsequently, Static and SMG JAWS II DC were washed and incubated (1 × 10^4^/well) with OT-II CD4^+^ TCH (5 × 10^4^/well) and (**a**) OVA323-339 or (**b**) OVA protein for an additional 24 hours in Static conditions. Culture supernatants were collected and assessed for IL-2 production by ELISA. Wells containing Static or SMG JAWS II DC and OT-II TCH without OVA323-339 or OVA protein were not included in the figure and yielded 0 cytokine upon assessment. (**c**) OT-I CD8^+^ T cells (5 × 10^4^/well) were added to Static and SMG BMDC (1 × 10^4^/well) with or without OVA257-264 for 24 hours in Static conditions. Culture supernatants were collected and the production of IFN-γ determined by ELISA. For all panels, the data represents the mean + SD of quadruplicates of two independent experiments. In (**a**,**b**), *p-value ≤ 0.05 comparing the activation of T cell IL-2 production by SMG and Static JAWS II DC as well as SMG and Static JAWS II DC (+cytokines). In (**c**), *p-value ≤ 0.05 comparing the activation of T cell IFN-γ production by SMG and Static BMDC, both including OVA257-264.

Next, we tested the hypothesis that SMG DC can activate T cells more effectively than Static DC. First, unstimulated JAWS II DC were cultured in SMG or Static conditions for 72 h and then co-cultured with OVA323-339-specific OT-II CD4^+^ T cell hybridomas (OT-II TCH) for 24 h. Both Static and SMG DC were capable of activating the OT-II TCH to produce IL-2 (Fig. 4a). However, T cells, activated by SMG JAWS II DC, produced a greater amount of IL-2 as compared to stimulation with Static JAWS II DC. Interestingly, SMG JAWS II DC could not promote T cell IL-2 production to the level generated by stimulation with mature Static DC (Static DC + cytokines). This suggests that the stress of SMG may not operate as a strong stimulant for maturation when compared to other agents, including the cytokine cocktail. Therefore, DC cultured in SMG acquire a maturation phenotype but to a lesser degree than achieved upon activation through cytokine stimulation and possibly TLR ligation.

In order to test the effect of SMG upon DC activation of CD8^+^ T cells, we employed BMDC rather than JAWS II DC, given the greater capacity of BMDC to take up exogenous antigen and load the peptide onto MHC class I molecules (i.e. cross-presentation)^32^. Thus, BMDC were incubated in SMG and Static conditions for 48 h, then OVA257-264-specific OT-I CD8^+^ T cells were added and culture continued for an additional 24 h in a Static environment. While both Static and SMG BMDC stimulated substantial production of IFN-γ by the CD8^+^ T cells, SMG BMDC augmented T cell cytokine production (Fig. 4c). Similar to SMG JAWS II DC, an increase in BMDC maturation markers correlated with an enhancement of the T cell response.

While the ability of SMG DC to present peptide and activate T cells has been tested in this study, we further sought to examine the capacity of DC, cultured in SMG and Static conditions, to process full length protein into peptides for the activation of T cells. JAWS II DC were cultured in SMG or Static conditions for 72 h and then OVA protein and OT-II TCH were added for the final 24 h of Static incubation. OVA323-339 must be enzymatically cleaved from the OVA protein, transported to the endocytic vesicle for loading onto the MHC class II molecules and then the MHC-peptide complexes displayed on the cell surface. A two-fold increase of IL-2 production by the OVA323-339-specific OT-II TCH was observed upon stimulation of the T cells with SMG JAWS II DC when compared to activation by Static DC (Fig. 4b). This could be due to SMG-mediated signaling, such as activation of the MAPK pathway, which can promote DC maturation, antigen processing and presentation. Recently, a member of the tumor necrosis factor ligand family, osteoprotegerin, induced MAPK signaling, which resulted in the expression of MHC class II molecules, CD80 and CD86 on the surface of monocytes^33^. This suggests that the antigen processing and/or presentation pathways of DC, and other antigen presenting cells, could be influenced by signals initiated upon SMG exposure. Currently, it is unclear if SMG affects the enzymatic activities of antigen processing pathways in the DC. Future work will investigate how SMG-mediated signaling may impact these pathways. Consequently, osteoprotegerin induced the production of IL-6 in human monocytes. Perhaps the IL-6 production of JAWS II DC was similarly influenced by MAPK signaling induced by SMG exposure. However, the increased T cell IL-2 from activation by unstimulated SMG JAWS II DC was nearly 4-fold less than that of T cells stimulated by cytokine-matured Static DC (Fig. 4b). This may be the product of numerous contributing pathways including STAT-3 that can be activated by ligation of the three cytokines, IFN-γ, IL-4 and TNF-α, to the corresponding receptors on the DC. Overall, SMG promotes a maturation phenotype in resting DC, as compared to Static DC, which leads to superior stimulation of T cell cytokine production. Yet, the increased T cell activity is significantly less than that of T cells stimulated by Static DC matured through common modalities, such as cytokine.

### Long-term culture in SMG diminishes JAWS II DC function

**Figure 5:**
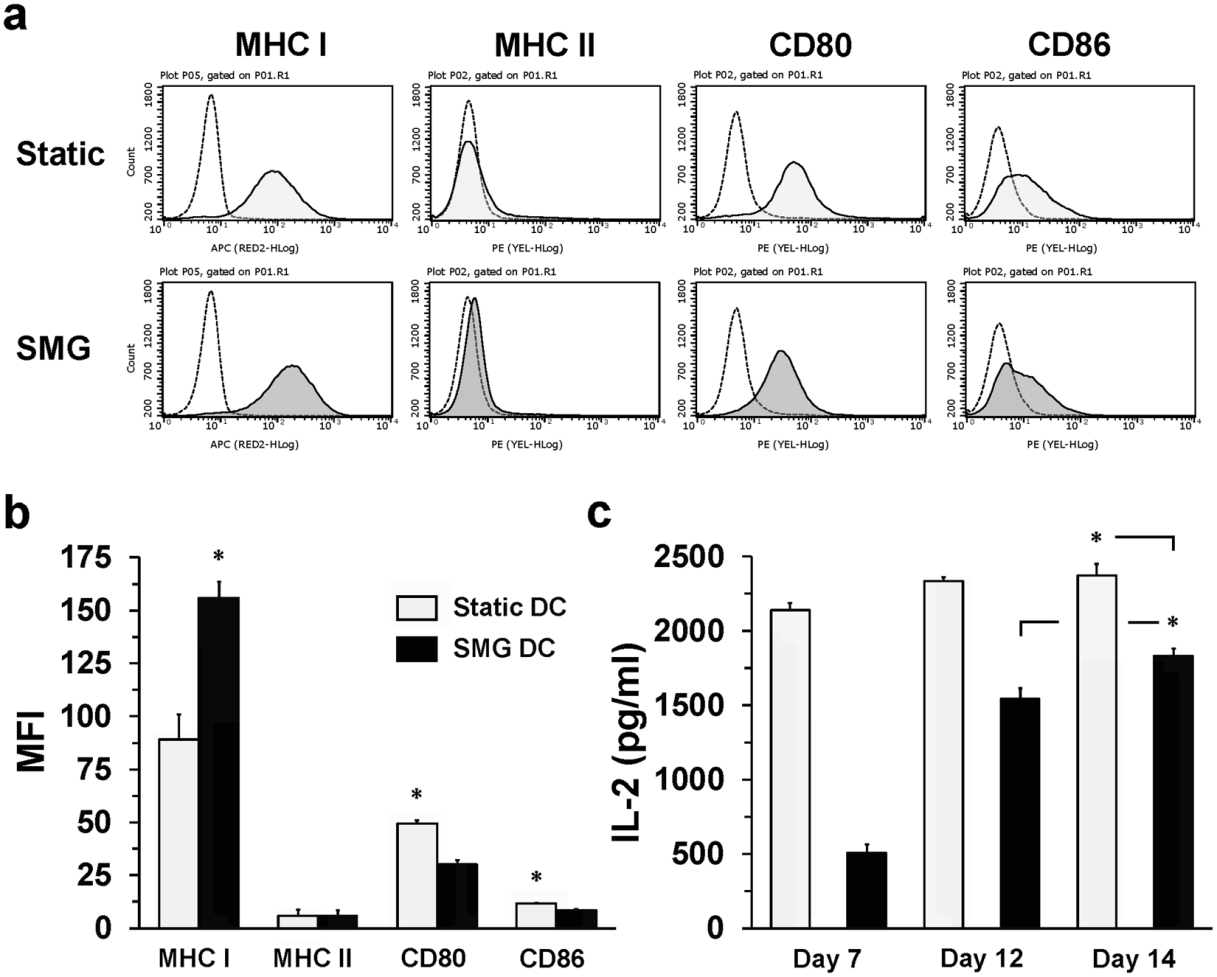
Long-term culture in SMG reduces the T cell stimulatory capacity of JAWS II DC. (**a**) JAWS II cells (2 × 10^5^/ml) were cultured in Static (light gray) or SMG (dark gray) conditions for 5 days. DC were collected and stained with antibodies for MHC class I and II, CD80 and CD86 and their corresponding isotype controls. Open histograms with broken lines represent isotype controls. (**b**) Graph represents the MFI of each of the surface molecules examined in (**a**) for Static (light gray bars) and SMG JAWS II DC (black bars). (**c**) JAWS II DC (2 × 10^5^/ml) were cultured in Static (light gray bars) or SMG (black bars) conditions for 7, 12 and 14 days. At each time point, Static and SMG JAWS II DC were harvested, washed and incubated with OT-II CD4^+^ TCH and OVA323-339 for an additional 24 hours in Static conditions. Culture supernatants were collected and assessed for IL-2 production by ELISA. One representative experiment of two independent experiments with similar results is shown for (**a**). The data in (**b**) represents the mean + SD of quadruplicates of two independent experiments. In (**b**), *p-value ≤ 0.05 comparing the expression of surface molecules by Static and SMG JAWS II DC. In (**c**), *p-value ≤ 0.05 comparing the activation of T cell IL-2 production by day 12 SMG and day 14 SMG JAWS II DC as well as day 14 Static and SMG JAWS II DC.

For astronauts, spontaneous dendritic cell maturation induced by microgravity could lead to a state of “exhaustion” in the DC population^34^. Such an impairment in the DC could reduce the reactivity of the T cell response to any antigen, thereby, creating immunodeficiency and a high risk for infection. Given that SMG promoted a maturation phenotype in DC, we examined the status of DC after a 5 d (120 h) incubation in either Static or SMG conditions. Unstimulated SMG JAWS II DC maintained significantly higher expression of MHC class I molecules on the surface when compared to Static DC (Fig. 5a,b). By contrast, expression of MHC class II molecules on the SMG DC were reduced compared to that of Static DC. In addition, CD80 and CD86 expression on SMG JAWS II DC were significantly reduced in comparison to Static DC. Despite higher MHC class I expression, SMG DC, with lower CD80 and CD86 on the surface, would likely be less effective at activating CD8^+^ T cells than Static DC. Thus, we expected that CD4^+^ T cell cytokine production should be greater in the presence of Static DC than SMG DC over longer periods of SMG exposure. To test this proposal, JAWS II DC were cultured in SMG or Static conditions for 7, 12 and 14 d, harvested and then incubated with OT-II TCH in Static conditions. Incubation of DC from 7–14 d in SMG had a significant impact upon T cell production of IL-2 when compared to Static controls (Fig. 5c). The greatest difference between T cell IL-2 production upon SMG and Static DC stimulation was 7 d with a nearly 2.5-fold reduction in cytokine. Surprisingly, IL-2 production by T cells stimulated with DC, that were cultured for 12 d in SMG, improved to more than half that of T cells activated by Static DC. Cytokine production increased significantly when the DC were cultured for 14 d in SMG. However, across all time points, IL-2 production by T cells, stimulated with Static DC, was significantly more than stimulation by SMG DC. This indicates that the DC may be “adapting” to the SMG environment rather than existing as exhausted cells. The molecular nature of the proposed adaptation of DC to the microgravity environment will be the focus of future work, given the importance of the information to the health of individuals of mission durations of months to years. Thus, while long-term SMG exposure diminishes dendritic cell functions, the percentages of IL-2 from T cells stimulated with SMG DC increases from 23% (7 d), 66% (12 d) and 77% (14 d) of that produced by the T cells activated with Static DC.

### Antigen processing and presentation by DC within SMG declines over time

**Figure 6:**
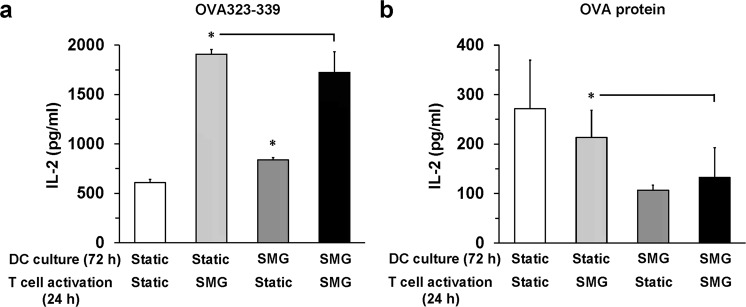
JAWS II DC can activate T cells within SMG but demonstrate a reduced capacity to capture and/or present antigens in long-term cultures. JAWS II DC (2 × 10^5^/ml) were cultured in Static (white and light gray bars) or SMG (dark gray and black bars) conditions for 72 hours. (**a**) Afterward, Static and SMG cultured JAWS II DC were harvested, washed and incubated with OT-II CD4^+^ TCH and OVA323-339 for an additional 24 hours in either Static or SMG conditions (10 ml total volume). (**b**) Harvested Static and SMG cultured JAWS II DC were refreshed with media and incubated with OT-II CD4^+^ TCH and OVA protein for an additional 24 hours in either Static or SMG conditions as in (**a**). Culture supernatants were collected and assessed for IL-2 production by ELISA. For (**a**,**b**), data represents the mean + SD of n = 10 and n = 6, respectively, of two independent experiments. In (**a**), *p-value ≤ 0.05 comparing the activation of T cell IL-2 production by SMG and Static DC in Static conditions as well as SMG and Static DC in SMG conditions. In (**b**), *p-value ≤ 0.05 comparing the activation of T cell IL-2 production by Static and SMG DC in SMG conditions.

We next assessed the ability of SMG and Static JAWS II DC to function in the activation of the OT-II TCH within SMG. DC were first incubated in either Static of SMG conditions for 72 h. Then both the T cells and OVA323-339 peptide were added to the DC and incubation continued another 24 h in either Static or SMG conditions. Static DC co-cultured with the T cells for 24 h in SMG repeated the increased IL-2 production by T cells as shown above when compared to the control (Fig. 6a, white and light gray bars). However, the amount of IL-2 produced by the T cells activated in SMG with Static DC was significantly more than that produced by T cells activated in the Static environment with SMG DC (Fig. 6a, light gray and dark gray bars). This striking difference in T cell IL-2 production is most likely due to the direct effect of the SMG environment upon the OT-II TCH. Previously, we demonstrated that exposure of the OT-II TCH to SMG for less than 72 h resulted in a more robust response to DC stimulation with significant production of IL-2 as compared to activated OT-II TCH in Static conditions^11^. Likely this extends to the heightened IL-2 production in wells where the JAWS II DC remains in the SMG environment (Fig. 6a, black bar) for the duration of the experiment (96 h). The difference between IL-2 levels in this culture and Static DC and T cells in SMG (Fig. 6a, light gray bar) is most likely a consequence of the period of DC exposure to SMG, 24 h vs 96 h. Thus, JAWS II DC, exposed to SMG for less than 72 h, acquired the capacity to promote increased T cell activation relative to Static DC, whether activation occurred within Static or SMG environments. Importantly, greater than 72 h exposure to SMG diminished JAWS II dendritic cell immunogenicity.

To address the question whether JAWS II DC were able to process and present antigen to activate the OT-II TCH, all within SMG, the DC were first incubated in either Static or SMG conditions for 72 h. SMG and Static DC were then incubated with OT-II TCH and OVA protein for an additional 24 h in either SMG or Static environments. Unexpectedly, the IL-2 production by T cells, upon stimulation with peptide-loaded Static DC, was similar regardless of whether activation occurred in Static or SMG conditions (Fig. 6b, white and light gray bars). Compared to the use of peptide (Fig. 6a, white and light gray bars), this suggests that SMG may disrupt pathways of antigen processing. This must be the case as a short-term exposure to SMG enhances DC signaling (Fig. 1), maturation (Fig. 2) and the capacity to activate T cells (Fig. 3). So why does short-term exposure to SMG lead to significant IL-2 production by T cells when peptide serves as the antigen source? Peptides can directly bind to MHC molecules on the surface of DC, unlike proteins that require internal enzymatic processing^35^. Longer exposure to SMG (72–96 h) intensified the reduction in T cell IL-2 production to statistical significance (Fig. 6, light gray, dark gray and black bars). This data further supports the idea that SMG negatively impacts antigen uptake and/or processing in DC, and potentially other antigen presenting cells. That is, the short-term augmentation of T cell sensitivity to antigen (Fig. 6a, light gray and black bars), that we have both previously reported and measured in this study, was no longer detected when DC processed OVA protein in SMG (Fig. 6b, black bar). DC did not recover antigen uptake and/or processing capacity since removal of the DC from SMG, at the time of OVA protein encounter, was unable to restore T cell IL-2 production to that of Static DC (Fig. 6b, white, light gray and dark gray bars). Therefore, SMG exposure of JAWS II DC, at time points beyond 72 h, negatively impacts antigen capture and/or processing, leading to a greater than 50% reduction in T cell responses as measured by IL-2.

## Conclusion

During the first 72 h of SMG exposure, cell signaling pathways are induced in JAWS II DC, but not in Static cultured DC. Despite the activation of STAT-5 and MAPK pathways, the numbers of SMG JAWS II DC are two-fold less than Static DC. This finding is consistent with a study involving 9–12 d culture of peripheral blood-derived human DC in an RCCS^36^. Following incubation, the numbers of Static cultured MHC II^+^ DC were nearly twice as many as those DC growing in the RCCS. When the cells were analyzed for the expression of maturation markers, Static DC have increased MHC class II and CD80 on the cell surface when compared to the SMG DC. Other markers examined, including CD86, were similarly expressed between Static and SMG DC. We showed that short-term SMG culture of DC resulted in an increased expression of maturation markers on DC. Given the above study assessed DC maturation after 9–12 d of RCCS culture, this information was unknown. The data reported here is consistent with their findings as long-term SMG culture of DC demonstrated similar reductions in the maturation proteins compared to the Static DC. Our data is further supported by the results from the 10-day Eneide mission to the International Space Station in 2005. DC, taken from the blood of one of the astronauts, were assessed for maturation status^8^. Post-flight DC expressed lower maturation markers than that of pre-flight DC.

We also demonstrated that long-term culture of DC in SMG resulted in reduced antigen uptake and/or processing for the activation of T cells. Similarly, human DC, cultured in the RCCS for 9–12 d, were unable to take up fungal conidia by phagocytosis to the extent of Static DC^36^. The same DC were assessed for cytokine production following culture in SMG and were found to produce markedly less than Static DC. We examined short-term cytokine production by the SMG JAWS II DC and demonstrated a robust level of cytokine compared to Static cultured DC. This increased cytokine accompanied the enhanced maturation status of SMG DC. However, long-term SMG culture diminished the maturation status of DC, and most likely, the cytokine production, albeit cytokine was not assessed in our cells.

Short-term SMG culture enabled DC to better activate T cells compared to Static DC, as measured by T cell IL-2 and IFN-γ production. IL-2, primarily a product of activated CD4^+^ T cells, plays a crucial role in the development of both CD4^+^ and CD8^+^ T cell responses^37^. During T cell activation, signal 3 cytokines from DC activate transcription factors within the T cells. Transcription factors induce gene expression associated with type-specific T cell responses. Moreover, the signal 3 cytokine, IL-12, induces phosphorylation and dimerization of STAT-4, which in turn, activates the transcription factor, T-bet. This factor induces IFN-γ production and additional gene expression, both associated with type 1 T cell development. Type 1 CD4^+^ T cells are termed T helper 1 (Th1) cells while type 1 CD8^+^ T cells are T cytotoxic 1 (Tc1) cells. Similarly, IL-4 from DC activates the transcription factor, GATA-3, through pSTAT-6. GATA-3 induces production of IL-4, IL-5 and IL-13 by differentiated T cells, designating the cells as type 2, Th2 and Tc2, cells. Th1/Tc1 cells are required for the elimination of intracellular viruses, pathogenic bacteria and tumors. Th2/Tc2 cells are important in the destruction of parasites and function in wound healing. IL-2 plays an important role in both type 1 and 2 responses by maintaining gene locus accessibility, providing cell survival during the activation process and upregulation of the involved transcription factors and signal 3 cytokine receptors. However, as our data demonstrates, long-term exposure of DC to SMG results in a reduction of CD4^+^ T cell IL-2 production. The loss of IL-2 production has been demonstrated during the course of several space flights using both assessment of astronaut serum levels and the activation of peripheral blood mononuclear cells (PBMC) by agent-mediated direct stimulation of activation pathways^38^. Together with our findings in this report and previously those concerning T cell activity in SMG, short-term exposure to SMG accentuates the functions of both DC and T cells, producing elevated IL-2 compared to Static cultures. Alternatively, exposure of DC and T cells to SMG more than 3 days results in diminished antigen activation of the T cells and subsequent reduced T cell responsiveness to the antigen. With mission durations greater than 3 days, the dampened IL-2 levels, upon T cell activation, must greatly impact overall immune health. As indicated above, the magnitude of the Th1 and Th2 response upon antigenic challenge will be significantly impaired, and thereby may account for the observed reactivation of latent pathogens, such as Epstein-Barr virus and cytomegalovirus, and the onset of opportunistic infections^39^. Indeed, IFN-γ production from splenocytes, derived from mice flown aboard the Space Shuttle (STS-131), was significantly lower than cytokine detected from activated ground splenocytes^40^. Similarly, reduced IL-4 gene expression was discovered from splenic tissues of mice flown for 13 days aboard the Space Shuttle (STS-135) compared to ground controls^41^. More importantly, IL-2 has a critical role in controlling the balance between T helper 17 (Th17) cells and T regulatory (Treg) cells^42^. In the absence of IL-2, Treg cells diminish greatly in number and function while Th17 cells expand to increase the potential for autoimmune disease and inflammatory disorders to develop. IL-6 and transforming growth factor-beta (TGF-β) signal for the differentiation of activated CD4^+^ T cells into Th17 effector cells, which secrete cytokines to promote the influx of phagocytic cells and inflammation. If Th17 cells are activated against self-peptides, then the inflammatory response can damage tissue and organs resulting in autoimmunity. IL-2 acts to suppress IL-6 receptor expression, and simultaneously signal through the IL-2 receptor on Treg cells that operate to inhibit immune responses to self-peptides. Overwhelming IL-6 production in the presence of microbial agents can promote Th17 cell differentiation as Treg cells consume the excess IL-2. However, IL-2 knockout animals develop systemic autoimmune disease due to the lack of production of Treg cells in the thymus. A reduction of IL-2 could potentially reduce Treg cell numbers and put the astronauts at a greater risk for autoimmunity or overt inflammatory conditions. Using a hind limb unloading mouse model, the numbers of Treg cells decreased and inflammatory mediators, such as IL-1, increased with the onset of colitis induced by dextran sulfate sodium^43^. Lastly, the expansion and effector functions of activated CD8^+^ T cells are highly dependent upon IL-2 production by Th cells. CD8^+^ T cells are important in the control and elimination of viral agents and neoplasms. Recently, murine CD8^+^ T cells (and CD4^+^ T cells) were cultured in a clinorotation bioreactor, similar to the RCCS, for up to 24 h^44^. The T cells, cultured in SMG, demonstrated reduced expression of the molecules associated with activation, CD25, CD69 and CD71, compared to Static control cells. Interestingly, the T cells were unable to proliferate to the extent of activated Static T cells similar to what we observed for the DC. IL-2 and IFN-γ production by the CD4^+^ and CD8^+^ T cells, respectively, were increased compared to Static cultured T cells, again as reported in this study. While concanavalin A, a plant mitogen, was used to activate the T cells above, we are currently examining the viability and function of antigen-specific CD8^+^ T cells in SMG using DC and peptide for natural stimulation of responses.

When JAWS II DC were cultured for periods >72 h in SMG, expression of the costimulatory molecules, CD80 and CD86 diminished, and IL-2 production by the antigen-specific T cells reduced significantly as well when compared to Static T cells. At the extended culture time of two weeks in SMG, the JAWS II DC began to stimulate more T cell IL-2 production than that obtained when DC were exposed to SMG between 5 and 7 d. However, the increased T cell reactivity remained significantly less than that of T cells stimulated by Static DC. When the T cells were activated within the SMG environment by JAWS II DC, exposed to less than 72 h of SMG, then IL-2 production was augmented compared to Static control cultures. However, DC exposure beyond 72 h greatly diminished antigen capture and/or processing and the activation of the T cells. Overall, OT-II TCH, interacting with antigen presenting JAWS II DC within an SMG environment less than 72 h, are functioning better than Static counterparts. DC and T cell exposure to SMG beyond 72 h results in a loss of functional capacity, thereby, creating the potential for immunodeficiency and unregulated immune responses in astronauts unless an unknown mechanism of adaptation is initiated as suspected by the results of long-term SMG culture of the DC.

## Data Availability

Protocols related to the harvest, culture and cryopreservation of OT-I CD8^+^ T cells and BMDC are previously published as referenced. Protocols related to culture of cells in the RCCS or flasks are discussed above and any further information can be made available by the corresponding author. JAWS II DC can only be obtained from ATCC. Otherwise, all relevant data are included in this article.
